# The efficacy and safety of setmelanotide in individuals with Bardet-Biedl syndrome or Alström syndrome: Phase 3 trial design

**DOI:** 10.1016/j.conctc.2021.100780

**Published:** 2021-05-03

**Authors:** Robert M. Haws, Gregory Gordon, Joan C. Han, Jack A. Yanovski, Guojun Yuan, Murray W. Stewart

**Affiliations:** a Marshfield Clinic Research Institute, Marshfield, WI, USA; b Rhythm Pharmaceuticals, Inc, Boston, MA, USA; c University of Tennessee Health Science Center, Memphis, TN, USA; d Children's Foundation Research Institute, Le Bonheur Children's Hospital, Memphis, TN, USA; e Section on Growth and Obesity, Eunice Kennedy Schriver National Institute of Child Health and Human Development, National Institute of Health, Bethseda, MD, USA

**Keywords:** Antiobesity drug, Appetite control, Obesity therapy, Phase III study

## Abstract

**Background:**

A phase 2 trial has suggested that treatment with the melanocortin-4 receptor (MC4R) agonist setmelanotide is associated with a decrease in hunger and weight-related outcomes in participants with Bardet-Biedl syndrome (BBS) and Alström syndrome. Here, we present the study design of an ongoing, randomized, double-blind, placebo-controlled, phase 3 trial to assess the long-term efficacy and safety of setmelanotide for the treatment of obesity and hyperphagia in individuals with BBS or Alström syndrome (ClinicalTrials.gov identifier: NCT03746522).

**Methods:**

It was initially planned that ~30 participants aged ≥6 years with a clinical diagnosis of BBS or Alström syndrome would be enrolled. Participants with obesity as defined by a body mass index ≥30 kg/m^2^ (in those aged ≥16 years) or a weight >97th percentile (in those aged 6–15 years) are included. Participants are initially randomized in a 1:1 ratio to receive setmelanotide or placebo for 14 weeks (period 1). Following period 1, all participants receive 38 weeks of open-label treatment with setmelanotide (period 2). In each treatment period, setmelanotide is administered at 3 mg once a day following completion of dose escalation. The primary endpoint is the proportion of participants aged ≥12 years achieving a clinically meaningful reduction from baseline (≥10%) in body weight after ~52 weeks (eg, following period 2). Safety and tolerability are assessed by frequency of adverse events.

**Conclusions:**

This pivotal trial is designed to evaluate the efficacy and safety of setmelanotide for the treatment of obesity and hyperphagia in individuals with BBS and Alström syndrome.

**Submission category:**

Study Design, Statistical Design, Study Protocols.

## List of abbreviations

AE: adverse events
BBS: Bardet-Biedl syndrome
CI: confidence interval
FAS: full analysis set
MC4R: melanocortin-4 receptor
QD: once a day

## 1 Introduction

The hypothalamic melanocortin-4 receptor (MC4R) is activated by neurons in the paraventricular nucleus and is involved in the regulation of metabolism and body weight [1,2]. The MC4R pathway is controlled by 2 opposing types of neurons [2,3]. Inhibition of the MC4R pathway is regulated by release of the inverse agonist agouti-related peptide from neuropeptide Y/agouti-related peptide neurons [3]. Activation of MC4R is dependent on melanocortin-stimulating hormone release by proopiomelanocortin neurons following activation of the leptin receptor [2].

Variants in genes involved in these pathways can result in Bardet-Biedl syndrome (BBS) and Alström syndrome, rare genetic diseases associated with impaired ciliary function and insufficient MC4R signaling [[4], [5], [6], [7]]. BBS is characterized by several major features, including rod-cone dystrophy, polydactyly, learning disabilities, hypogonadism, and renal anomalies [8]. Alström syndrome is characterized by similar features, including cone-rod dystrophy, hearing loss, type 2 diabetes mellitus, dilated cardiomyopathy, and hepatic and renal abnormalities [9]. Additionally, BBS and Alström syndrome are both characterized by early-onset obesity and hyperphagic behaviors likely due at least in part to defects in the MC4R signaling pathway [[10], [11], [12], [13]].

Setmelanotide is a melanocortin agonist that acts as a substitute for α-melanocortin–stimulating hormone at MC4R-activating neurons [14,15]. Treatment with setmelanotide may help overcome many of the effects of genetic deficiencies that occur upstream of MC4R in the pathway. In phase 2 and 3 clinical trials, setmelanotide treatment resulted in reduced body weight and hunger scores in individuals with obesity due to proopiomelanocortin and leptin receptor deficiency [14,15]. In a recent phase 2 trial of 8 individuals with BBS and 4 with Alström syndrome, setmelanotide was associated with a decrease in hunger and weight-related outcomes for up to 12 months [16,17].

BBS is a rare genetic disease of obesity, with a global prevalence ranging from 1 in 3700 to 1 in 160,000 individuals, and with higher prevalence rates being observed in isolated populations [18]. Clinical trial design for rare disease has novel challenges additional to those associated with trial design for common diseases [19]. Although a randomized controlled trial design is considered the gold standard for evaluating therapeutic efficacy, the large number of participants required is often not feasible to recruit for rare diseases such as BBS [[20], [21], [22]]. Recruiting individuals with rare diseases can be challenging because of a lack of disease awareness, diagnostic challenges, and the overall rarity of the disease [19]. Insufficient available research on the natural history of the disease can make determining relevant outcome measures, efficacy endpoints, and follow-up duration challenging [19,20]. In addition, interindividual heterogeneity and the smaller study size of rare disease trials can complicate the study analysis. These factors highlight the importance of clear and well-designed trials tailored for rare diseases.

Here, we present the study design of an ongoing, randomized, double-blind, placebo-controlled, phase 3 trial with an open-label extension to assess the long-term efficacy and safety of setmelanotide for the treatment of obesity and hyperphagia in individuals with BBS or Alström syndrome (ClinicalTrials.gov identifier: NCT03746522).

## 2 Methods

### 2.1 Study design and participants

This is a randomized, placebo-controlled, double-blind, phase 3 trial with an open-label extension evaluating the efficacy and safety of setmelanotide in individuals with BBS or Alström syndrome. The trial is being conducted at ~10 centers worldwide. Participants aged ≥6 years with a clinical diagnosis of BBS (according to Beales criteria) [8] or Alström syndrome (according to Marshall criteria) [9] are eligible (Table 1). All participants with Alström syndrome must have a genetically confirmed diagnosis at the time of enrollment. Although genetic testing is common in individuals with BBS, diagnosis of BBS is based on meeting clinical criteria [8]. A small proportion of participants with BBS (≤10%) may be enrolled in the study without prior genetic testing to assess whether previous genetic testing changes the patient population in a manner that might affect responsiveness to setmelanotide. It was initially planned that the trial would enroll ~30 participants, including at least 20 with BBS and 6 with Alström syndrome, with a maximum of 6 participants aged <12 years being initially enrolled, including ~4 with BBS and 2 with Alström syndrome. Recruitment of the primary cohort was completed in 2019, with 38 participants (32 with BBS and 6 with Alström syndrome) being enrolled.

**Table 1:** Diagnostic criteria for BBS and Alström syndrome.

	Requirement	Primary/major features	Secondary/minor features
BBS [8]	4 primary features or 3 primary and 2 secondary features	• Rod-cone dystrophy • Polydactyly • Obesity • Learning disabilities • Hypogonadism in males • Renal anomalies	• Speech disorder/delay • Strabismus/Cataracts/Astigmatism • Brachydactyly/Syndactyly • Developmental delay • Polyuria/Polydipsia (nephrogenic diabetes insipidus) • Ataxia/poor coordination/imbalance • Mild spasticity (especially lower limbs) • Diabetes mellitus • Dental crowding/hypodontia/small roots/high arched palate • Left ventricular hypertrophy/congenital heart disease • Hepatic fibrosis
Alström syndrome [9] (in those aged 6 to ≤14 years)	2 major criteria or 1 major and 3 minor criteria	• Mutation of *ALMS1* and/or family history of Alström syndrome • Vision (nystagmus, photophobia, diminished acuity)^a^	• Obesity and/or insulin resistance and/or T2DM • History of DCM/CHF • Hearing loss • Hepatic dysfunction • Renal failure • Advanced bone age
Alström syndrome [9] (in those aged ≥15 years)	2 major and 2 minor criteria or 1 major and 4 minor criteria	• Mutation of *ALMS1* and/or family history of Alström syndrome • Vision (history of nystagmus in infancy/childhood, legal blindness, cone-rod dystrophy by ERG)	• Obesity and/or insulin resistance and/or T2DM • History of DCM/CHF • Hearing loss • Hepatic dysfunction • Renal failure • Short stature • Hypogonadism^b^ • Irregular menses and/or hyperandrogenism^c^

BBS, Bardet-Biedl syndrome; CHF, congestive heart failure; DCM, dilated cardiomyopathy; ERG, electroretinography; T2DM, type 2 diabetes mellitus.

a If old enough for testing: cone dystrophy by ERG.

b In males.

c In females.

Participants with obesity as defined by a body mass index ≥30 kg/m^2^ (in those aged ≥16 years) or weight >97th percentile (in those aged 6–15 years) are included. Those receiving an intensive diet or exercise regimen with or without the use of weight loss agents that has resulted in >2% weight loss within the last 2 months are excluded. Additionally, those using any medication approved to treat obesity within 3 months of randomization (eg, orlistat, lorcaserin, phentermine-topiramate, naltrexone-bupropion) are also excluded. Additional key inclusion and exclusion criteria are shown in Table 2.

**Table 2:** Key inclusion and exclusion criteria.

Inclusion criteria	• Clinical diagnosis of BBS [8] or Alström syndrome [9]
	• ≥6 years of age^a^
	• BMI ≥30 kg/m^2^ (participants aged ≥16 years) or weight >97th percentile for age and sex (participants aged 6–15 years)
Exclusion criteria	• Recent (within 2 months) intensive diet and/or exercise regimen that has resulted in >2% weight loss
	• Use of any medication that is approved to treat obesity within 3 months of randomization
	• Prior gastric bypass surgery resulting in weight loss of >10% with no clear evidence of weight regain
	• HbA_1c_ >9.0% at screening
	• Close family (ie, participant, parents, or siblings) history of skin cancer or melanoma, or history of oculocutaneous albinism
	• Significant dermatologic findings relating to melanoma or premelanoma skin lesions

BBS, Bardet-Biedl syndrome; BMI, body mass index; HbA_1c_, glycated hemoglobin.

a It was planned that a maximum of 6 participants under the age of 12 years would be initially enrolled into the pivotal cohort.

This trial is being conducted in accordance with the International Council on Harmonisation for Good Clinical Practice, Declaration of Helsinki, and appropriate regulatory requirements. The trial is being conducted only at sites in which institutional review board approval has been obtained. Written informed consent from participants or guardians prior to participation is required.

### 2.2 Procedures and assessments

**Fig. 1:**
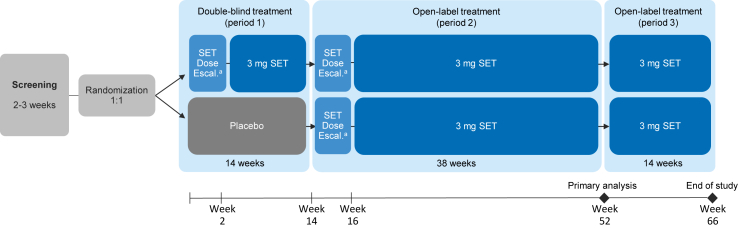
Schematic for the design of the overall study. Escal., escalation; QD, once a day; SET, setmelanotide. ^a^During dose escalation, participants who are ≥16 years of age receive setmelanotide 2 mg QD for 2 weeks, which increases to 3 mg at the beginning of week 3; participants who are <16 years of age receive setmelanotide 1 mg QD for the first week, 2 mg for the second week, and 3 mg at the beginning of week 3.

The trial has 3 treatment periods (Fig. 1). Participants are initially randomized in a 1:1 ratio to receive setmelanotide or placebo for 14 weeks (period 1). Participants who are ≥16 years of age and randomized to setmelanotide receive a subcutaneous injection of setmelanotide 2 mg once a day (QD) during a 2-week dose escalation, which increases to 3 mg at the beginning of week 3. Participants who are <16 years of age and randomized to setmelanotide initially receive a subcutaneous injection of setmelanotide 1 mg QD for the first week, which increases to 2 mg for the second week and to 3 mg at the beginning of the third week. Similarly, matching placebo is administered via a subcutaneous injection in those randomized to placebo.

Following period 1, all participants receive 38 weeks of open-label treatment with setmelanotide (period 2). To maintain the blind, an upward dose escalation to a fixed dose of 3 mg of setmelanotide is performed during the first 2 weeks of period 2. This dose escalation follows the same procedure as that for period 1 and is performed independently of the initial treatment received. The primary analysis is performed following period 2. Following period 2, participants continue to receive open-label setmelanotide for another 14 weeks (period 3).

Participants are assessed via site visits during period 1 at the beginning of weeks 1, 3, 7, and 11; during period 2 at the beginning of weeks 15, 17, 23, 29, 35, 41, 47, and 53; and during period 3 at the beginning of week 60 and at the end of the study. At each visit, the study drug is dispensed; body weight, height, vital signs, and concomitant medications are reviewed. Adverse events (AEs) are monitored throughout the study. Laboratory parameters are recorded at each visit during period 1 and 2 and at the end of study. Additional body composition measurements, including body fat, are recorded at screening and at the beginning of week 53.

All participants who are able to self-assess their hunger complete a daily hunger questionnaire in the morning prior to a morning meal and dosing. For participants ≥12 years of age, the assessments use a Likert-type scale, where 0 = not hungry at all and 10 = hungriest possible, to generate a hunger score. Participants are asked to provide scores for morning hunger, most hunger (ie, hunger at their hungriest point in the past 24 h), and average hunger over the past day. For participants between 6 and < 12 years of age, morning hunger is assessed using a pictorial (smiley face) version with scores ranging from 0 to 4, with 0 = not hungry at all and 4 = hungriest possible.

During weeks 2 and 15, AEs are assessed, and concomitant medications are reviewed via a telephone call. Changes in depression and suicidality are assessed and monitored using the Columbia Suicide Severity Rating Scale and the Patient Health Questionnaire-9 over the entire course of the trial.

### 2.3 Randomization and blinding

On the first day of period 1, participants who remain eligible are assigned a unique randomization number indicating the initial treatment assignment based on a code that is generated prior to the start of the study. Randomization is stratified by age group (≥12 vs < 12 years of age) and disease (BBS vs Alström syndrome). The study remains blinded to participants, caregivers, and assessors through the end of period 2. Placebo and setmelanotide are identical in appearance and supplied in identical packaging. At each visit during period 1, the study pharmacist selects the correct treatment using a package code and provides the blinded study medication to the participant or caregiver; following period 1, the study pharmacist provides open-label setmelanotide. Unblinding only occurs in the event of a medical emergency where the identity of the study drug may be necessary to appropriately treat the participant. If a participant is unblinded, the reason and timing of unblinding is documented. Of note, it may be difficult to fully maintain treatment blinding as tanning and hyperpigmentation can be very noticeable in patients receiving setmelanotide treatment.

### 2.4 Study endpoints

The primary endpoint is the proportion of participants aged ≥12 years achieving a reduction from baseline (≥10%) in body weight after ~52 weeks of treatment with setmelanotide (ie, following period 2). For participants randomized to the setmelanotide group, baseline is defined as the last available measurement prior to the randomization. For participants randomized to the placebo group, baseline is defined as the last available measurement prior to the first dose of open-label setmelanotide treatment.

Key secondary efficacy endpoints include percent change in body weight and hunger scores after ~52 weeks of treatment with setmelanotide and proportion of participants with ≥25% improvement in hunger score after ~52 weeks of treatment with setmelanotide in participants aged ≥12 years. A complete list of primary and secondary efficacy endpoints is included in Table 3.

**Table 3:** Study efficacy endpoints.

Primary efficacy endpoint	Timing	Study population
Proportion of participants who achieve ≥10% reduction from baseline in body weight	~52 weeks of treatment^a^	FAS among those who are ≥12 years of age at baseline
Key secondary endpoints		
Mean percent change from baseline in body weight	~52 weeks of treatment^a^	FAS among those who are ≥12 years of age at baseline
Mean percent change in weekly average of daily hunger score	~52 weeks of treatment^a^	FAS among those who are ≥12 years of age at baseline
Proportion of participants who achieve ≥25% reduction in weekly average of daily hunger score	~52 weeks of treatment^a^	FAS among those who are ≥12 years of age at baseline
Other secondary endpoints		
Mean percent change from baseline in body weight compared with placebo	Week 14	PCS among those who are ≥12 years of age at baseline
Mean percent change in weekly average of daily hunger score compared with placebo	Week 14	PCS among those who are ≥12 years of age at baseline

FAS, full analysis set; PCS, 14-week placebo-controlled analysis set.

a Treatment with setmelanotide. For participants randomized to the setmelanotide group, baseline is defined as the last available measurement prior to the randomization; for participants randomized to the placebo group, baseline is defined as the last available measurement prior to the first dose of open-label setmelanotide treatment.

Exploratory endpoints include various body composition/metabolic outcomes, safety/tolerability, and changes in depression or suicidality as assessed by the Columbia Suicide Severity Rating Scale and Patient Health Questionnaire-9. Safety and tolerability are assessed by frequency of AEs. All AEs are graded using the National Cancer Institute Common Terminology Criteria for Adverse Events.

### 2.5 Statistical analyses

Efficacy is assessed primarily in the full analysis set (FAS; Table 3), which includes all participants who received at least 1 dose of study drug and have baseline data. The primary analysis is conducted in the FAS among those who are ≥12 years of age at baseline. Safety is assessed in the safety analysis set, which comprises participants who received at least 1 dose of study drug or placebo. Efficacy analyses are performed according to randomization, and safety analyses are performed according to treatment received. No interim analyses are planned.

The primary statistical hypothesis is that the proportion of participants treated with setmelanotide for ~52 weeks who achieve ≥10% reduction from baseline in body weight is greater than a historical control rate of 10% in the FAS among those who are ≥12 years of age at baseline. Data from the Clinical Registry Investigating Bardet-Biedl Syndrome (ClinicalTrials.gov identifier: NCT02329210) was used to provide input for the sample size/power calculations. A sample size of 7 participants provides ~91% power at 1-sided alpha of 0.025 to yield a statistically significant difference, assuming a 66% response rate in participants treated with setmelanotide. This suggests that powering the study for the primary endpoint requires <10 participants; the size of the trial also considers the rarity of BBS and Alström syndrome and a desire to better understand the effect of setmelanotide in these individuals. Thus, ~30 participants (including 6 participants with Alström syndrome) are planned to be enrolled in the study; this number is suitable for a single pivotal trial to support the indications in BBS and Alström syndrome and to provide robust information for both the between-group analysis in period 1 and the comparison to the historical control rate in period 2.

The primary endpoint is assessed using an exact binomial test at a 1-sided significance level of 0.025, which was chosen on the basis of the small sample size. A 2-sided 95% confidence interval (CI) is calculated using the exact Clopper-Pearson method. The statistical criterion for rejection of the null hypothesis corresponds to the 2-sided 95% CI for setmelanotide of the response rate excluding 10% (ie, the lower bound of the 95% CI > 0.10). Because of the rarity of the disease indication and small sample size, no multiplicity adjustments are being performed for key secondary endpoints. The *P*-values and the corresponding CIs are being provided. All AEs and discontinuations due to AEs are being summarized descriptively with frequencies and percentages.

For participants randomized to the setmelanotide group, baseline for statistical analyses following ~52 weeks of treatment is defined as the last available measurement prior to the randomization. For participants randomized to the placebo group, baseline is defined as the last available measurement prior to the first dose of open-label setmelanotide treatment. For participants randomized to the placebo arm with <52 weeks of setmelanotide treatment by the timing of the primary analysis at the end of period 2, multiple imputation is used to impute measurements after ~52 weeks of treatment for the primary analysis.

## 3 Discussion

Hyperphagia can be an overwhelming burden to both the individual and the family, severely affecting health, quality of life, and finances. Environmental controls, such as supervising children around food, securing food sources, reducing energy intake, and adhering to meal schedules, are essential management strategies for weight regulation [23]. However, these controls do not address the persistent hyperphagia [24], which can negatively affect quality of life and social dynamics.

Individuals with BBS and Alström syndrome can experience hyperphagia leading to obesity, and no treatments have been commercially approved to manage these symptoms in these individuals. This is the first pivotal phase 3 clinical trial to investigate the safety and efficacy of an MC4R agonist for the treatment of obesity and hyperphagia in individuals with BBS or Alström syndrome. Because of the rarity of BBS and Alström syndrome, a traditional trial design is not being applied, and the primary endpoint does not directly compare the placebo and treatment arms. However, an initial placebo-controlled period is included in the trial design, which allows some secondary endpoints to be assessed as a comparison between treatment and placebo.

This trial includes a 14-week randomized, double-blind placebo period allowing for evaluation of any placebo effect associated with setmelanotide, which is often difficult to achieve in rare disease clinical trials [19], Additionally, all participants receive open-label setmelanotide treatment following the randomized placebo period, which can alleviate ethical concerns of placebo delivery in rare diseases as well as ease reluctance for enrollment [20].

BBS is known to be associated with variants in >20 genes, with variants in *BBS1, BBS2,* and *BBS10* representing approximately half of the total BBS cases [25]. There are known genotype-phenotype relationships in patients with BBS [25], and response to setmelanotide could be dependent on genotype. Additional analyses from this trial could also assess response to setmelanotide by genetic background.

This study is designed to evaluate the efficacy and safety of setmelanotide for the treatment of obesity and hyperphagia in individuals with BBS or Alström syndrome. This study aims to build upon the results of a phase 2 study that suggested setmelanotide may reduce hunger and body weight in individuals with BBS and Alström syndrome. If successful, the results from this pivotal trial may support the use of setmelanotide for treatment of obesity and hyperphagia in individuals with BBS or Alström syndrome.

## Role of the funding source

The sponsor of the study (Rhythm Pharmaceuticals, Inc) designed the trial with assistance from academic investigators. The sponsor aided in data collection, data analysis, data interpretation, and writing of the report. All authors had final responsibility for the decision to submit for publication.

## Data statement

Because this article presents a clinical study design, no data are currently available to share.

## Funding source

This study was supported by Rhythm Pharmaceuticals, Inc.

## Declaration of competing interest

RMH is a consultant for Rhythm Pharmaceuticals, Inc and Trinity Life Sciences and receives grant funding from the Bardet-Biedl Syndrome Foundation.

GG, GY, and MWS are employed by and may own stock in Rhythm Pharmaceuticals, Inc.

JCH has received grant support for clinical investigations from the Memphis Research Consortium, Le Bonheur Children's Foundation Research Institute, and Rhythm Pharmaceuticals, Inc.

JAY receives grant support for clinical investigations from the NICHD, NIH, Soleno Therapeutics Inc, and Rhythm Pharmaceuticals, Inc.
